# Transcriptomic Differences Between Human Trabecular Meshwork Stem Cells and Trabecular Meshwork Cells Reveal Specific Biomarker Profiles

**DOI:** 10.3390/cimb47070514

**Published:** 2025-07-03

**Authors:** Rong Du, Ajay Kumar, Enzhi Yang, Jingxue Zhang, Ningli Wang, Yiqin Du

**Affiliations:** 1 Beijing Institute of Ophthalmology, Beijing Tongren Eye Center, Beijing Tongren Hospital, Beijing Key Laboratory of Intelligent Diagnosis Technology and Equipment for Optic Nerve-Related Eye Diseases, Capital Medical University, Beijing 100730, China; rongdu@usf.edu (R.D.); jingxuezh@ccmu.edu.cn (J.Z.); 2 Department of Ophthalmology, Morsani College of Medicine, University of South Florida, Tampa, FL 33612, USA; eyang@usf.edu; 3 Department of Ophthalmology, University of Pittsburgh, Pittsburgh, PA 15213, USA; ajaykumar@niper.ac.in

**Keywords:** trabecular meshwork, stem cells, transcriptomic profiling, RNA sequencing, microarray, hub genes, glaucoma

## Abstract

Glaucoma is a leading cause of irreversible blindness, normally associated with dysfunction and degeneration of the trabecular meshwork (TM) as the primary cause. Trabecular meshwork stem cells (TMSCs) have emerged as promising candidates for TM regeneration toward glaucoma therapies, yet their molecular characteristics remain poorly defined. In this study, we performed a comprehensive transcriptomic comparison of human TMSCs and human TM cells (TMCs) using RNA sequencing and microarray analyses, followed by qPCR validation. A total of 465 differentially expressed genes were identified, with 254 upregulated in TMSCs and 211 in TMCs. A functional enrichment analysis revealed that TMSCs are associated with development, immune signaling, and extracellular matrix remodeling pathways, while TMCs are enriched in structural, contractile, and adhesion-related functions. A network topology analysis identified *CXCL3*, *CXCL6*, and *BMP2* as robust TMSC-specific hub genes, and *LMOD1* and *BGN* as TMC-specific markers, with expression patterns confirmed by qPCR. These findings define distinct molecular signatures of TMSCs and TMCs, providing reliable biomarkers for cell identity and a foundation for future stem cell-based therapies targeting TM dysfunction in glaucoma.

## 1. Introduction

The human trabecular meshwork (TM) is a specialized tissue located at the anterior chamber angle of the eye, responsible for draining up to 90% of aqueous humor and thereby regulating intraocular pressure [1,2,3,4]. In primary open-angle glaucoma, dysfunction or loss of TM cells (TMCs) leads to reduced outflow facility and elevated intraocular pressure, contributing to optic nerve damage and vision loss [5,6,7,8]. Approaches to restore or replace reduced TMCs are of great interest in glaucoma research. Recent studies suggest that the TM contains a population of stem cells—termed trabecular meshwork stem cells (TMSCs)—which reside in the tissue and can give rise to functional TMCs [9,10,11,12,13,14]. These TMSCs, identified in human TM tissue, exhibit characteristics of mesenchymal stem cells, reflecting the developmental origin of the TM. They have demonstrated multipotency in vitro, being capable of differentiating into multiple cell lineages, including functional TMCs. Further, human TMSCs were able to home in on the normal mouse TM region without immunorejection after xenotransplantation [15], specifically homing in on the laser-damaged mouse TM region [16] and the TM of mice with MYOC mutation [17] to regenerate the TM and improve the outflow facility to reduce intraocular pressure for glaucoma treatment. It emphasizes the importance of distinguishing TMSCs and TMCs for cell-based therapies for glaucoma.

The identification of specific biomarkers that distinguish TMSCs from TMCs is important for both basic research and potential clinical applications. Such markers can validate the identity and purity of cultured cells and serve as readouts for the successful differentiation of TMSCs into functional TMCs. Traditional TM cell markers include several genes highly expressed in the TM relative to other ocular tissues—examples include aquaporin-1 (AQP1), chitinase 3-like 1 (CHI3L1), matrix Gla protein (MGP), and myocilin (MYOC) [18,19,20,21]. These markers have been used collectively to identify TMCs, as no single definitive TM marker exists. Likewise, TMSCs are known to express various stem cell-associated markers such as CD90 (Thy-1), CD73, ABCG2, and NESTIN, and pluripotency or neural crest markers including OCT4, KLF4, and HNK-1 [9,11,16]. Despite this knowledge, the comprehensive molecular differences between TMSCs and TMCs have not been fully delineated. Most previous studies have focused on a limited number of canonical markers, lacking systematic transcriptomic comparisons between the two cell types. Moreover, few studies have integrated multiple high-throughput platforms or validated candidate markers across independent techniques and biological replicates. As a result, reliable and reproducible biomarkers that can robustly distinguish TMSCs from mature TMCs remain insufficiently defined, which hinders both mechanistic studies of TM regeneration and the development of standardized protocols for cell-based glaucoma therapies.

High-throughput transcriptomic technologies provide a powerful approach to profiling global gene expression differences between cell types [22]. Microarray analyses have been used historically to examine TM cell gene profiles and identify candidate markers, while RNA sequencing (RNA-seq) offers a more recent, sensitive method to capture the full transcriptome, including low-abundance transcripts and novel genes or isoforms [23,24,25,26]. Combining these platforms can increase confidence in the results by cross-validating the findings and leveraging the strengths of each method.

In this study, we performed a comprehensive comparative analysis of gene expression in human TMSCs versus primary cultured TMCs using both microarray and RNA-seq. By directly comparing the transcriptomic profiles from these two approaches, we aimed to identify robust differentially expressed genes that serve as distinguishing biomarkers for TMSCs and TMCs. We also validated key differences at the mRNA level using quantitative PCR (qPCR) to ensure that the observed transcriptomic variations are reproducible and biologically relevant. The insights gained from this work will improve our understanding of TM stem cell biology and provide valuable molecular tools for assessing TM cell identity and guiding TM cell replacement strategies in glaucoma therapy.

## 2. Materials and Methods

### 2.1. Cell Culture and Sample Preparation

Human TMSCs were cultured and passaged as previously described [10,27]. In brief, de-identified human donor corneas unsuitable for transplantation were obtained from the Center for Organ Recovery and Education (Pittsburgh, PA, USA) or Lions Eye Bank (Tampa, FL, USA). After removal of the iris and ciliary processes, a circumferential incision was made along the anterior margin of Schwalbe’s line, and the TM tissue was carefully dissected. The isolated TM tissue was quartered and placed into 25 cm^2^ culture flasks for explant culture in a growth medium for 10–14 days. The culture medium consisted of Opti-MEM (Invitrogen, Carlsbad, CA, USA) supplemented with 5% fetal bovine serum (FBS; Thermo Fisher, Waltham, MA, USA), 10 ng/mL epidermal growth factor (EGF; Sigma-Aldrich, St. Louis, MA, USA), 100 µg/mL bovine pituitary extract (Life Technologies, Carlsbad, CA, USA), 20 µg/mL ascorbic acid, 200 µg/mL calcium chloride, 0.08% chondroitin sulfate (Sigma-Aldrich), 100 IU/mL penicillin, 100 µg/mL streptomycin, and 50 µg/mL gentamicin (Thermo Fisher). After initial outgrowth, the cells were detached using TrypLE (Invitrogen), seeded at a density of 500–1000 cells/cm^2^ to promote clonal expansion, and passaged at 70–80% confluence. The phenotypic characterization of TMSCs was performed via immunofluorescence staining, with positive expression of stem/progenitor markers NESTIN and OCT4, confirming their stem-like identity.

Human TMCs were cultured using a similar explant method but maintained in Dulbecco’s Modified Eagle’s Medium (DMEM)/F12 supplemented with 10% FBS [10,28,29]. The cells were passaged when they reached 100% confluence. TM cell identity was confirmed by responsiveness to dexamethasone treatment with increased MYOC expression and expression of TMC markers including CHI3L1, which are associated with extracellular matrix regulation in mature TM tissue.

The donors had no history of glaucoma and were between 33 and 69 years of age. Each corneoscleral rim was processed within 5 days post-mortem to isolate cells. Detailed donors’ information from which the cells were isolated and cultured is listed in Table 1. Both TMSC and TMCs were used between passages 3 and 5. Each experiment was repeated with at least three biological and technical replicates.

### 2.2. RNA Isolation

Total RNA was extracted from TMSC and TMC cultures for RNA-seq, microarray, and qPCR experiments. The cells were washed with phosphate-buffered saline (PBS, Gibco, Grand Island, NY, USA), followed by lysis in culture dishes using an RLT buffer (Qiagen, Venlo, Netherlands). RNA isolation was performed using the RNeasy Mini Kit (Qiagen) according to the manufacturer’s instructions. The RNA pellet was treated with Ambion RNase-free DNase in DNase I buffer (Invitrogen). RNA quantity was assessed by spectrophotometry (NanoDrop, Thermo Fisher). Only high-quality RNA samples (A_260/280_~1.8–2.0, RNA Integrity Number > 7) were used for downstream analyses.

### 2.3. RNA Seq and Analysis

RNA-seq was performed to obtain a high-resolution profile of gene expression differences. The RNA pellet was dissolved in RNase-free diethyl pyrocarbonate (DEPC) water and sent to GENEWIZ, LLC. (South Plainfield, NJ, USA) for sequencing.

After sequencing, raw reads were demultiplexed and quality-checked using FastQC (v0.12.0). Adapter sequences and low-quality bases were trimmed with Fastp, applying a quality score threshold of 20 and a minimum read length of 50. Cleaned reads were then aligned to the human reference genome, GRCh38, using HISAT2, a splice-aware aligner. The resulting BAM files were sorted, indexed, and assessed for alignment metrics, including the percentage of mapped reads and read distribution across exons and introns. Gene-level expression counts were generated using featureCounts with aligned BAM files and an Ensembl gene annotation file. The raw counts for each gene were imported into R for further analysis, where they were normalized for library size, and variance stabilization was applied for downstream analysis.

DESeq2 (version 1.46.0) was used to perform a differential expression analysis between TMSCs and TMCs, calculating fold changes and Wald test *p*-values for each gene. Genes with a false discovery rate-adjusted *p*-value < 0.05 and a fold-change cutoff (≥1 or ≤−1) were identified as significantly differentially expressed. RNA-seq also enabled the detection of transcripts not represented on microarrays, as well as insights into transcript isoforms and non-coding RNAs. A list of differentially expressed genes (DEGs) was compiled, annotated with gene names and functions for downstream interpretation. A Gene Ontology (GO) enrichment analysis was conducted to identify significant biological processes, cellular components, and molecular functions, while a Kyoto Encyclopedia of Genes and Genomes (KEGG) pathway analysis highlighted enriched signaling pathways associated with the DEGs. A volcano plot was generated to visually represent the distribution of genes based on log_2_ fold changes and adjusted *p*-values, distinguishing TMSC-upregulated, TM-upregulated, and non-significant genes.

To identify hub genes in the TMSC- and TMC-enriched gene networks, we first constructed protein–protein interaction (PPI) networks using the STRING database (version 11.5). DEGs upregulated in either TMSCs or TMCs were imported into the STRING platform, with a confidence score cutoff of 0.4. The resulting interaction files were then imported into Cytoscape (version 3.9.1) for network visualization and topological analysis.

Hub genes were identified using the CytoHubba plugin (v3.9.1) based on four centrality algorithms: Maximum Neighborhood Component (MNC), Edge Percolated Component (EPC), Closeness Centrality, and Degree Centrality. Each method independently ranked the top 20 genes based on their connectivity or influence within the network. The intersecting hub genes across all four algorithms were identified using a Venn diagram constructed with the VennDiagram package (v1.7.3) in R (version 4.2.0). Shared genes were considered the most robust hub candidates for each cell type.

### 2.4. Microarray Gene Expression Profiling

RNA samples were extracted from TMSC and TMCs using the previously described method and processed for microarray analysis with the Affymetrix Human Genome U133 Plus 2.0 Array platform (Applied Biosystems, Foster City, CA, USA). The raw data (CEL files for Affymetrix) were imported into gene expression analysis software, where background correction and normalization were conducted using the Robust Multi-array Average (RMA) algorithm. A differential expression analysis was performed with the Limma package (v3.52.4) in R, incorporating empirical Bayes moderation of variance. A contrast matrix was created to compare the TMSC to the TMCs. Genes with a *p*-value < 0.05 (adjusted for false discovery rate) and an absolute log_2_ fold change ≥ 2.0 were considered significantly differentially expressed.

### 2.5. Quantitative Real-Time PCR Validation

Total RNA was extracted from cultured cells using the RNeasy Mini Kit following lysis in the RLT buffer, as described above. RNA concentration and purity were assessed using a NanoDrop spectrophotometer. Complementary DNA (cDNA) was synthesized from 500 ng of total RNA using SuperScript III Reverse Transcriptase (Invitrogen) according to the manufacturer’s protocol, with random hexamer primers.

qPCR was performed using PowerUp SYBR Green Master Mix (Thermo Fisher) on a StepOnePlus Real-Time PCR System (Applied Biosystems). Primers for target genes were either designed using the NIH Primer-BLAST tool or adopted from previously published studies. The primer sequences and references are listed in Table 2. Each reaction was performed in a 20 μL volume containing 2 μL of diluted cDNA, 10 μL of 2× SYBR Green Master Mix, and 0.75 μM of each forward and reverse primer.

The housekeeping gene *18S rRNA* was used as an internal normalization control due to its stable expression across TMSC and TMC samples, as we previously reported [10,11]. Negative controls without template cDNA were included in each run to exclude contamination or primer dimer artifacts. The thermocycling conditions were as follows: initial denaturation at 95 °C for 2 min, followed by 40 cycles of 95 °C for 15 s and 60 °C for 1 min. A melt curve analysis was performed to confirm the amplification specificity.

Relative gene expression levels were calculated using the 2^−ΔΔCt^ method. All qPCR assays were conducted using three independent biological replicates derived from three different human donors, with each reaction performed in technical triplicates.

### 2.6. Immunofluorescent Staining

Cultured cells were fixed with 4% paraformaldehyde for 15 min at room temperature, permeabilized with 0.5% Triton X-100 for 10 min, and blocked with 1% bovine serum albumin (BSA) for 1 h. The cells were incubated overnight at 4 °C with primary antibodies against NESTIN, OCT4, and CHI3L1. After three washes with PBS, appropriate fluorescent secondary antibodies and DAPI were applied for 2 h at room temperature.

For tissue staining, 10 μm cryosections of human trabecular meshwork tissue were used. The sections were fixed in 4% paraformaldehyde, permeabilized with 0.5% Triton X-100, and blocked with 1% BSA for 1 h at room temperature. Slides were incubated with primary antibodies against AQP1, ABCG2, ANKG, CHI3L1, NESTIN, NCAM, and MUCIN1 for 48 h at 4 °C. After thorough PBS washes, corresponding fluorescent secondary antibodies and DAPI were applied for 4 h at room temperature. The slides were then washed five times, mounted with antifade medium, and imaged using the Keyence BZ-X800 fluorescence microscope.

For Whole-Mount Staining, following fixation, the posterior segment of the eye was removed by making a circular incision approximately 1.5 mm posterior to the limbus. The anterior segment, including the cornea and trabecular meshwork, was carefully dissected to remove the lens, iris, and ciliary body and then cut into four equal quadrants. The tissues were incubated with primary antibodies against MGP, AQP1, and MUCIN1, followed by appropriate fluorescent secondary antibodies. Nuclear counterstaining was performed with DAPI at room temperature for 30 min. After five washes in PBS, the samples were mounted on glass slides for imaging. Stitched image stacks were acquired using a confocal laser scanning microscope via sequential scanning, using FV10-ASW Viewer software (v4.2, Olympus Corporation, Tokyo, Japan).

### 2.7. Data Analysis and Statistics

Statistical analyses were performed using one-way or two-way ANOVA followed by Tukey’s multiple comparisons test in GraphPad Prism (v10.2.2, San Diego, CA, USA). A *p*-value < 0.05 was considered statistically significant.

## 3. Results

### 3.1. Isolation and Characterization of TMSCs and TMCs

**Figure 1:**
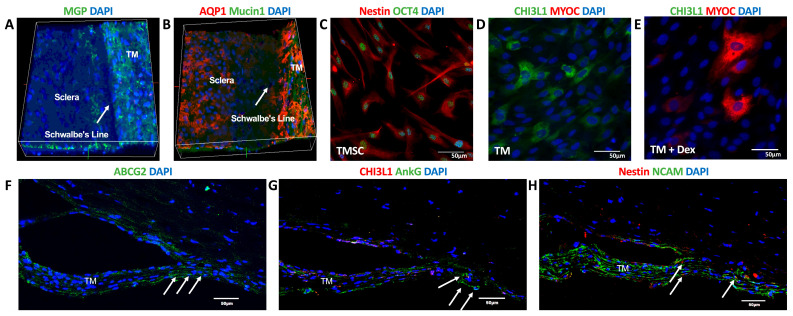
Isolation, culture, and characterization of trabecular meshwork stem cells (TMSCs) and trabecular meshwork (TM) cells. (**A**–**C**) Whole-mount immunofluorescence staining of the trabecular meshwork region showing expression of MGP (green, **A**), AQP1 (red, **B**), MUCIN1 (green, **B**) with DAPI nuclear counterstaining (blue), confirming the identity of the TM region. White arrows in (**A**) and (**B**) indicate Schwalbe’s line. (**C**) Immunofluorescence staining of cultured TMSCs showing stem cell markers NESTIN (red) and OCT4 (green), counterstained with DAPI (blue). (**D**,**E**) Staining of cultured TM cells without (**D**) and with (**E**) dexamethasone treatment showing expression of CHI3L1 (green) and MYOC (red), with DAPI (blue). (**F**–**H**) Immunofluorescence staining of human corneal tissue sections showing the main locations of cells positive to stem cell marker ABCG2 (green, **F**), ankyrin G (green, **G**), NESTIN (red, **H**), or TM cell marker CHI3L1 (red, **G**) and NCAM (green, **H**). White arrows in (**F**) indicate ABCG2-positive cells; in (**G**), ankyrin G–positive cells; and in (**H**), NESTIN-positive cells. Nuclei were counterstained with DAPI (blue). Images in (**A**,**B**,**E**–**G**) were captured at 10× magnification; (**C**–**E**) were captured at 40× magnification. Scale bar = 50 µm.

To verify the successful isolation and identity of TMSCs and TMCs, we performed immunofluorescence staining on whole-mount tissues, cultured cells, and TM tissue sections (Figure 1).

In whole-mount staining of the TM region, matrix Gla protein (MGP) [30] was strongly expressed, confirming the anatomical identity of the trabecular meshwork (Figure 1A). The co-localization of aquaporin-1 (AQP1, red) [18] and MUCIN1 (green) [10] further delineated the TM region (Figure 1B). Cultured TMSCs exhibited strong immunoreactivity for the stem/progenitor markers NESTIN (red) [10] and OCT4 (green) [10], consistent with their stem-like phenotype (Figure 1C). In contrast, cultured TMCs were positive for CHI3L1 (green), a marker associated with differentiated TMCs (Figure 1D) [10,31]. TMCs expressed MYOC after treatment with 100nM dexamethasone for 5 days (Figure 1E) [29].

In tissue sections, the putative stem cell marker ABCG2 (green) was detected within the TM region with more prominence in the Schwalbe’s line region, where the arrows point in Figure 1F. The co-expression of CHI3L1 (red) and ankyrin-G (ANKG, green) [10] further confirmed the regional identity of TMCs (Figure 1G). Additionally, NESTIN (red) was mainly present in the Schwalbe’s line region, indicating stem cell location, and neural cell adhesion molecule (NCAM, green) [32] was present in the TM, indicating the different locations of TMSCs and TMCs (Figure 1H).

Collectively, these results validate that the isolated and cultured TMSCs and TMCs possess distinct phenotypic characteristics. TMSCs exhibit a molecular signature consistent with stemness, while TMCs express markers indicative of terminal differentiation. This verification was essential to ensure the accuracy and biological relevance of subsequent transcriptomic comparisons using microarray and RNA-seq.

### 3.2. Differential Gene Expression by RNA-Seq

**Figure 2:**
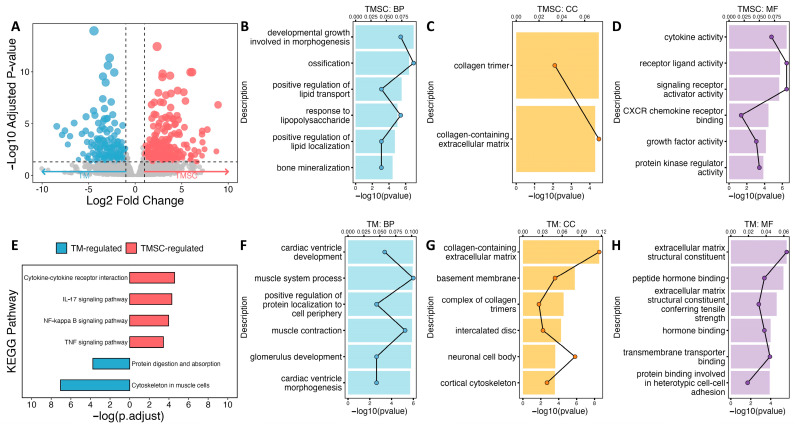
Transcriptomic analysis of trabecular meshwork stem cells (TMSCs) and trabecular meshwork (TM) cells. (**A**) Volcano plot displaying differentially expressed genes (DEGs) between trabecular meshwork stem cells (TMSCs) and trabecular meshwork (TM) cells. (**B**–**D**) Gene Ontology (GO) enrichment analysis of TMSC-enriched DEGs, including biological process (BP) (**B**), cellular component (CC) (**C**), and molecular function (MF) (**D**). (**E**) KEGG pathway enrichment in TMSC and TM cells. (**F**–**H**) GO enrichment analysis of TM-enriched DEGs, including BP (**F**), CC (**G**), and MF (**H**).

RNA-seq revealed substantial transcriptomic differences between TMSCs and TMCs. Using a threshold of absolute log_2_ fold change ≥ 1 and an adjusted *p*-value < 0.05, we identified a total of 465 DEGs (Figure 2A). Among them, 254 genes were significantly upregulated in TMSCs, while 211 genes were upregulated in TMCs.

A GO enrichment analysis demonstrated that TMSCs were significantly enriched in biological processes associated with developmental growth, morphogenesis, ossification, lipid transport, bone mineralization, and response to lipopolysaccharide (Figure 2B). Enriched cellular components included collagen trimers and the collagen-containing extracellular matrix (Figure 2C), indicating a prominent role for TMSCs in extracellular matrix remodeling. In terms of molecular functions, TMSCs exhibited increased enrichment in cytokine activity, receptor–ligand binding, signaling receptor activation, CXCR chemokine receptor binding, growth factor activity, and protein kinase regulator activity (Figure 2D). These findings suggest that TMSCs are functionally engaged in tissue development, inflammatory signaling, and growth factor-mediated regulation, consistent with their regenerative and progenitor roles.

In contrast, GO terms enriched in TMCs were primarily related to muscle and cardiac system development, including cardiac ventricle morphogenesis, muscle contraction, glomerulus development, and the regulation of protein localization to the cell periphery (Figure 2F). Enriched cellular components included the collagen-containing extracellular matrix, basement membrane, collagen trimers, intercalated discs, neuronal cell bodies, and cortical cytoskeleton (Figure 2G). A molecular function analysis showed the overrepresentation of terms such as extracellular matrix structural constituent, peptide hormone binding, tensile strength conferring activity, hormone binding, transmembrane transporter binding, and protein binding involved in heterotypic cell–cell adhesion (Figure 2H). These annotations emphasize the functional specialization of TMCs in contractile behavior, extracellular matrix architecture, and intercellular adhesion, reflecting their mature phenotype and role in maintaining intraocular pressure homeostasis.

A KEGG pathway analysis further supported the divergent biological roles of the two cell types (Figure 2E). TMSCs showed significant enrichment in immune- and inflammation-related signaling pathways, including cytokine–cytokine receptor interaction, IL-17 signaling, NF-κB signaling, and TNF signaling pathways. These results point to an active immunomodulatory role in TMSCs. In contrast, TMCs were enriched in pathways associated with cytoskeletal organization and metabolic activity, such as actin cytoskeleton regulation in muscle cells, and protein digestion and absorption. Together, these findings highlight the immunological and developmental responsiveness of TMSCs and the structural and metabolic specialization of TMCs.

### 3.3. Identification of TMSC- and TMC-Specific Hub Genes

To identify key functional regulators within the transcriptomic networks of TMSCs and TMCs, we conducted a hub gene analysis based on the upregulated DEGs in each group using Cytoscape. A topological evaluation was performed using four distinct centrality algorithms: Maximum Neighborhood Component (MNC), Edge Percolated Component (EPC), Closeness centrality, and Degree centrality.

**Figure 3:**
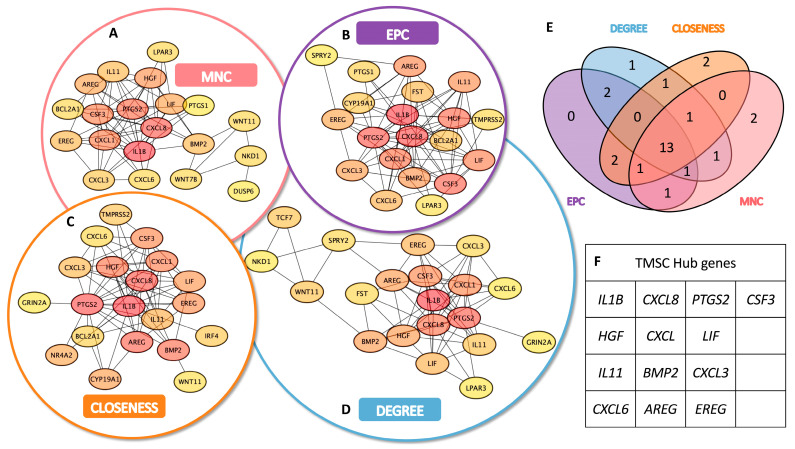
Identification of hub genes in TMSCs based on PPI network topology analysis. (**A**–**D**) Top 20 hub genes ranked by four centrality algorithms in the protein–protein interaction (PPI) network of upregulated genes in TMSCs: (**A**) Maximum Neighborhood Component (MNC), (**B**) Edge Percolated Component (EPC), (**C**) Closeness centrality, and (**D**) Degree centrality. Each algorithm highlights a partially overlapping but distinct set of candidate hub genes. (**E**) Venn diagram showing the overlap among the top 20 genes identified by the four algorithms. Thirteen genes were consistently ranked as hub genes by all methods. (**F**) List of the 13 consensus hub genes identified in TMSCs, representing the most centrally connected regulators within the TMSC-specific gene interaction network.

**Figure 4:**
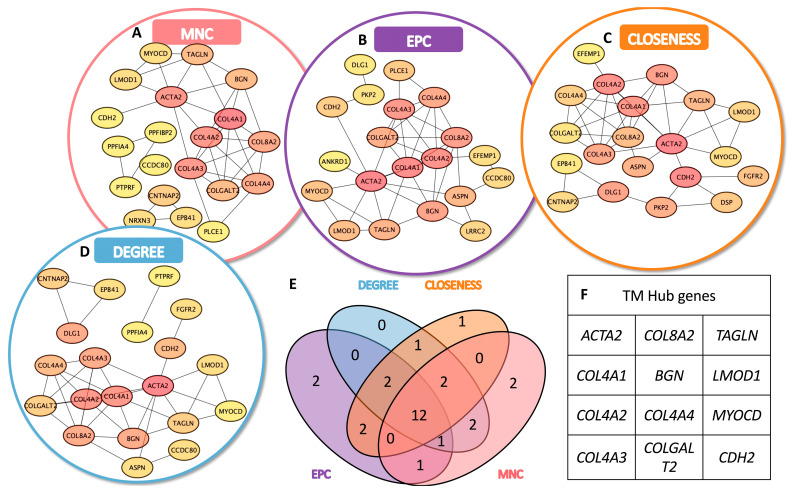
Identification of hub genes in TMCs based on PPI network topology analysis. (**A**–**D**) Top 20 hub genes ranked by four centrality algorithms in the PPI network of upregulated genes in TMCs: (**A**) MNC, (**B**) EPC, (**C**) Closeness, and (**D**) Degree. Each method emphasizes different aspects of node centrality in the network. (**E**) Venn diagram illustrating the intersection among hub genes identified by the four algorithms. Twelve genes were shared across all methods and defined as robust hub genes in TMCs. (**F**) List of the 12 consensus hub genes in TMCs, representing key regulatory nodes potentially involved in extracellular matrix organization, mechanotransduction, and TM-specific function.

In the TMSC group, each algorithm produced a ranked list of the top 20 hub genes, with partial overlap among methods (Figure 3A–D). A similar analysis was applied to TMC-upregulated DEGs (Figure 4A–D). A Venn diagram analysis identified a set of 13 consensus hub genes in TMSCs (Figure 3E,F) and 12 consensus hub genes in TMCs (Figure 4E,F), defined as genes commonly ranked in the top 20 by all four algorithms.

These intersecting hub genes represent the most centrally connected and potentially critical regulators within each cell-type-specific network. They offer promising candidates for further mechanistic studies and may serve as reliable molecular markers to distinguish stem-like TMSCs from differentiated TMCs.

### 3.4. Microarray Analysis Confirms Transcriptomic Distinctions

**Figure 5:**
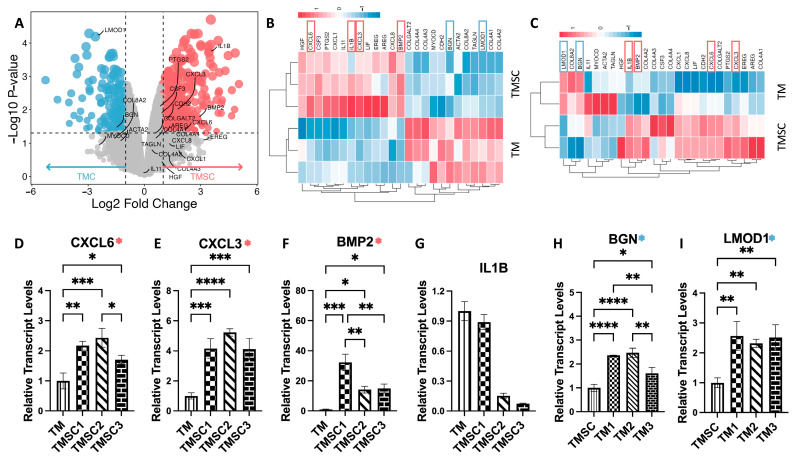
Cross-platform validation and qPCR confirmation of hub gene expression between TMSCs and TMCs. (**A**) Volcano plot of differentially expressed genes in TMSCs versus TMCs from microarray data. Red: upregulated in TMSCs; blue: upregulated in TMCs. (**B**,**C**) Heatmaps showing the expression profiles of 25 hub genes identified through RNA-seq (**B**) and microarray (**C**), demonstrating clear separation between TMSC and TMC samples, and confirming consistency across platforms. (**D**–**G**) qPCR validation of *CXCL3*, *CXCL6*, and *BMP2*, which were significantly upregulated in TMSCs, and *IL1B*, which showed no significant difference between the two groups. (**H**,**I**) qPCR validation of *LMOD1* and *BGN*, which were significantly upregulated in TMCs and serve as representative markers of the TMC group. Throughout the figure, pink represents TMSCs and blue represents TMCs. All qPCR results are shown as mean ± SD (n = 3 biological replicates from independent TMSC and TMC derived from different donors); *p* < 0.05 was considered statistically significant. * *p* < 0.05; ** *p* < 0.01; *** *p* < 0.001; **** *p* < 0.0001.

To validate and complement the RNA-seq results, we performed parallel transcriptomic profiling using high-density Affymetrix microarrays on an independent set of TMSC and TMC samples. Following RMA normalization and quality control, a differential expression analysis was conducted. Genes with an adjusted *p*-value < 0.05 and an absolute log_2_ fold change ≥ 2.0 were considered significant. Based on these criteria, 363 genes were significantly upregulated and 211 genes were downregulated in TMSCs relative to TMCs (Figure 5A). Figure 5B shows a heatmap of the RNA-seq of 25 hub genes, while Figure 5C shows a heatmap of a microarray of 25 hub gens.

Heatmaps of the 25 previously identified hub genes further demonstrated the consistency between platforms. Figure 5B displays RNA-seq-based expression patterns of these hub genes, while Figure 5C shows their corresponding microarray profiles. In both datasets, a clear separation between TMSC and TMC expression signatures was observed.

Notably, several TMSC-enriched hub genes identified in RNAseq—such as *IL1B*, *CXCL3*, *BMP2*, and *CXCL6*—also exhibited significant upregulation in the microarray analysis, reinforcing their association with the stem-like phenotype. Similarly, TMC-upregulated genes including *LMOD1* and *BGN* demonstrated consistent overexpression across both platforms.

An overview of these validated genes, including their full names and primary biological functions, is presented in Table 3 (referenced from GeneCards: https://www.genecards.org/, accessed on 10 April 2025). These results underscore the robustness of the observed transcriptomic distinctions between TMSCs and TMCs and support the use of these genes as candidate biomarkers for cell identity.

### 3.5. qPCR Validation of Key Hub Genes

To experimentally validate the hub genes identified by RNA-seq and microarray, we performed qPCR on cultured human TMSCs and TMCs, isolated using the same criteria described in Figure 1. As shown in Figure 5D–I, the expression levels of six selected hub genes were examined to assess consistency with transcriptomic data.

*CXCL3*, *CXCL6*, and *BMP2* showed significantly higher expression in TMSCs compared to TMCs, with average fold changes of 4.50, 2.10, and 20.49, respectively, aligning with both RNA-seq and microarray results. Conversely, *LMOD1* and *BGN* were validated as TMC-enriched genes, with fold changes of 2.47 and 2.15, respectively. Interestingly, *IL1B*, although initially identified as a TMSC-specific hub gene, did not exhibit a statistically significant difference between the two groups in qPCR validation. These results support the reliability of most transcriptomic findings while highlighting the importance of multi-platform confirmation.

## 4. Discussion

In this study, we systematically compared the transcriptomic profiles of human TMSCs and primary cultured TMCs to uncover distinct molecular characteristics and identify reliable cell-type-specific biomarkers. Through integrated RNA-seq and microarray analyses, we identified 465 differentially expressed genes, among which 254 were upregulated in TMSCs and 211 in TMCs. Gene Ontology and KEGG analyses revealed that TMSCs were enriched in pathways related to development, immune signaling, and extracellular matrix remodeling, whereas TMCs were associated with structural, contractile, and adhesion-related functions. The network topology analysis using four centrality algorithms identified 13 TMSC-specific and 12 TMC-specific hub genes, where *CXCL3*, *CXCL6*, and *BMP2* (TMSCs), and *LMOD1*, *BGN*, and *IL1B* (TMCs) were consistently validated across RNA-seq and microarray platforms. qPCR validation confirmed the differential expression of most hub genes, although *IL1B* did not show significant differences, highlighting the importance of multi-platform confirmation. Together, these results delineate a robust transcriptional distinction between stem cells and functional differentiated TM cell populations.

We observed that TMSCs exhibit a transcriptomic profile characterized by genes involved in tissue development, immune regulation, and extracellular matrix (ECM) interaction. This is consistent with their progenitor status and regenerative potential, as TMSCs were previously reported to be immunosuppressive without inducing rejections after xenotransplantation and to be able to remodel the TM tissue [15,16,17]. Notably, the enrichment of biological processes such as morphogenesis, ossification, and lipid transport reflects the capacity of TMSCs to participate in tissue remodeling and homeostasis. Furthermore, the upregulation of pathways related to cytokine activity and growth factor signaling—such as CXCL chemokines and BMP family members—highlights the paracrine communication functions of TMSCs that may influence surrounding cells in the TM microenvironment.

In contrast, TMCs displayed a gene expression pattern reflective of terminal differentiation and functional specialization. Genes involved in muscle system development, contractility, and ECM structural maintenance were enriched in this group, which aligns with the known biomechanical and barrier functions of the TM in regulating aqueous humor outflow [2,33]. The pronounced expression of cytoskeletal components, basement membrane proteins, and adhesion molecules in TMCs underscores their role in maintaining tissue architecture and mechanical integrity under constant intraocular pressure fluctuation [34,35,36].

Our identification and validation of hub genes further emphasize these distinctions. *CXCL3* and *CXCL6*, markedly upregulated in TMSCs, are CXC motif chemokines that not only mediate immune cell recruitment but may also serve autocrine roles in supporting stemness and migration [37]. *BMP2*, another TMSC-enriched gene, is a canonical regulator of osteogenic and mesenchymal differentiation, which may indicate a latent multilineage potential in TMSCs [38,39]. These findings support the concept that TMSCs are not merely a quiescent reserve but may actively respond to injury or stress via cytokine- and growth factor-mediated signaling.

In contrast, *LMOD1* and *BGN* were confirmed as TMC-specific genes. *LMOD1* is a regulator of actin filament elongation, commonly associated with contractile tissues [40,41]. Its high expression in TMCs suggests a role in cytoskeletal organization, potentially contributing to the mechanotransduction properties of the TM tissue [42,43]. *BGN*, a small leucine-rich proteoglycan, is essential for collagen fibrillogenesis and ECM stiffness regulation—both of which are altered in glaucomatous TM tissues or mechanosensitive insults of pressure and stretch [44,45]. Thus, their elevated expression in TMCs likely reflects their participation in the structural maintenance of outflow resistance.

Interestingly, *IL1B*, which was upregulated in both the RNA-seq and microarray datasets, failed to show significant differential expression in qPCR validation. This highlights an important caveat in transcriptomic studies: mRNA levels are influenced by both technical and biological factors, including donor variability, sample handling, and the dynamic nature of inflammation-related genes. Given *IL1B*’s known role in mediating oxidative stress and ECM degradation, its expression may be temporally regulated or context-specific, rather than constitutively elevated in stem-like states [46].

Functionally, the identified gene sets may serve as useful markers to monitor TMSC differentiation status, to optimize TM cell-derived therapeutic products, or to define pathological shifts in glaucomatous tissue. From a translational perspective, leveraging these markers could improve the fidelity of cell-based models and guide the development of stem cell therapies aimed at restoring TM structure and function and lowering intraocular pressure in glaucoma. These transcriptomic findings reinforce the therapeutic promise of TMSCs observed in preclinical glaucoma models [9,10,11,12,13,14]. By defining gene signatures linked to stemness and paracrine signaling, our study supports future TMSC-based strategies focusing on TM regeneration and intraocular pressure reduction.

Future work should focus on the mechanistic roles of the validated hub genes in TM physiology and pathology. Functional studies, including gene knockdown or overexpression, and in vivo validation in animal models, will be essential to establish causality and therapeutic relevance. Furthermore, the immunomodulatory properties suggested by the TMSC signature—particularly involving cytokine and chemokine signaling—warrant investigation in the context of inflammation-mediated TM dysfunction.

In conclusion, our integrated transcriptomic and experimental analysis delineates distinct molecular landscapes of TMSCs and TMCs, identifies robust biomarkers, and provides insight into the cellular programs underlying TM maintenance and regeneration. These findings contribute to the growing understanding of TM biology and may facilitate the development of novel diagnostic and therapeutic strategies for glaucoma.

## 5. Conclusions

In summary, this study provides a comprehensive transcriptomic comparison between human TMSCs and mature TMCs, integrating RNA-seq, microarray, and qPCR validation to identify robust cell-type-specific biomarkers. TMSCs exhibited gene expression signatures associated with developmental signaling, cytokine activity, and regenerative potential, whereas TMCs were enriched for genes involved in cytoskeletal structure, extracellular matrix organization, and tissue stability. A network-based hub gene analysis further highlighted key regulatory genes, including CXCL3, CXCL6, and BMP2 in TMSCs, and LMOD1 and BGN in TMCs, with consistent validation across platforms. These findings not only elucidate the molecular identities of TMSCs and TMCs but also provide a set of candidate markers that may facilitate cell characterization, quality control, and future development of TM-targeted regenerative therapies for glaucoma.

## Figures and Tables

**Table 1:** Donors’ information.

Cell Type	Age	Gender	Source	Ethnicity	Method
TMSC1	43	Female	Human	Caucasian	RNA-seq
TMSC2	61	Male	Human	Caucasian	RNA-seq
TMSC3	52	Female	Human	African American	RNA-seq
TM1	47	Male	Human	Caucasian	RNA-seq
TM2	69	Female	Human	African American	RNA-seq
TM3	55	Male	Human	Caucasian	RNA-seq
TMSC4	39	Male	Human	Caucasian	Microarray
TMSC5	59	Female	Human	Caucasian	Microarray
TM4	33	Male	Human	African American	Microarray
TM5	48	Female	Human	Caucasian	Microarray
TMSC6	51	Male	Human	Caucasian	qPCR
TMSC7	62	Female	Human	African American	qPCR
TMSC8	58	Male	Human	Caucasian	qPCR
TM6	45	Female	Human	Caucasian	qPCR
TM7	54	Male	Human	African American	qPCR
TM8	60	Female	Human	Caucasian	qPCR

**Table 2:** Primer sequences used for qPCR in this study.

Gene Name	DNA Sequence
*18S rRNA*	Forward: CCCTGTAATTGGAATGAGTCCAC
	Reverse: GCTGGAATTACCGCGGCT
*LMOD1*	Forward: CTTCCCTTGAGTGCCAGTGA
	Reverse: AGGTCATCCATGAGCAGCCT
*BMP2*	Forward: CGGTGAGGTCAGCTTCACAT
	Reverse: GGCCACAACTCCTCCAATCA
*BGN*	Forward: GATGGCAAAGGTGGCTAAGGAC
	Reverse: TACTGCAGTCCCTCCATGAATGT
*CXCL3*	Forward: CTTGACAGGAACGTCCGCAG
	Reverse: ACAGGGCACTTTGTCTTGGT
*CXCL6*	Forward: AGCTAACAGTCATTACGAGCCT
	Reverse: GACAGCTTTCCGGACTTCACT
*IL1B*	Forward: AACCTCTTCGAGGCACAAGG
	Reverse: GGCGAGCTCAGGTACTTCTG

**Table 3:** Gene symbols, full names, and functional roles of hub genes.

	Gene Symbol	Full Name	Function
TMSC	*IL1B*	Interleukin 1 Beta	Encodes a pro-inflammatory cytokine involved in inflammation, cell proliferation, cell proliferation, differentiation, apoptosis, and immune responses.
	*CXCL3*	C-X-C Motif Chemokine Ligand 3	Encodes a chemokine that attracts neutrophils and plays a role in inflammation.
	*BMP2*	Bone Morphogenetic Protein 2	Encodes a TGF-beta ligand critical for bone and cartilage development.
	*CXCL6*	C-X-C Motif Chemokine Ligand 6	Encodes a chemokine that attracts neutrophils and has antibacterial properties.
TM	*LMOD1*	LIM And Cysteine Rich Domains 1	Encodes a LIM-domain protein involved in protein–protein interactions.
	*BGN*	Biglycan	Encodes a proteoglycan that regulates bone growth, muscle development, collagen fibril assembly, and immunity.

## Data Availability

The data presented in this study are available from the corresponding author Y.D. on request.
